# National Trends and Disparities in the Incidence of Hepatocellular Carcinoma, 1998–2003

**Published:** 2008-06-15

**Authors:** Faruque Ahmed, Joseph F Perz, Patricia M Jamison, Carol Friedman, Beth P Bell, Sandy Kwong

**Affiliations:** Centers for Disease Control and Prevention; Centers for Disease Control and Prevention, Atlanta, Georgia; Centers for Disease Control and Prevention, Atlanta, Georgia; Centers for Disease Control and Prevention, Atlanta, Georgia; Centers for Disease Control and Prevention, Atlanta, Georgia; California Department of Health Services, Sacramento, California

## Abstract

**Introduction:**

Previous studies indicate that the incidence of hepatocellular carcinoma in the United States is increasing. These reports, however, have contained limited information on population groups other than whites and blacks.

**Methods:**

We assessed recent incidence rates and trends for hepatocellular carcinoma by using newly available national data from cancer registries participating in the Centers for Disease Control and Prevention's National Program of Cancer Registries and the National Cancer Institute's Surveillance, Epidemiology, and End Results Program. Data from registries in 38 states and the District of Columbia met our criteria; these data covered 83% of the U.S. population. We computed age-adjusted incidence rates and annual percentages of change from 1998 through 2003.

**Results:**

The registries that we used reported 48,048 cases of hepatocellular carcinoma (3.4 cases per 100,000 population per year) for the study period. Whites accounted for three-fourths of cases. The incidence rate for blacks was 1.7 times higher than that for whites, and the rate for Asians/Pacific Islanders was 4 times higher than that for whites. Hispanics had 2.5 times the risk of non-Hispanics. Among Asian/Pacific Islander subgroups, rates were highest for people of Vietnamese and Korean origin. For all races/ethnicities combined, the annual percentages of change were 4.8% for males and 4.3% for females (*P* < .05). The annual percentage of change was highest for people aged 45–59 years (9.0%, *P* < .05). The annual percentage of change for Asians/Pacific Islanders was statistically unchanged.

**Conclusion:**

We document rising incidence rates of hepatocellular carcinoma in the United States during a time when the overall incidence of cancer has stabilized. Efforts to collect representative etiologic data on new hepatocellular carcinoma cases are needed to enable better characterization of trends and to guide the planning and evaluation of prevention programs.

## Introduction

Approximately 18% of new cases of cancer worldwide are attributable to infection. In developing countries, the proportion is 26%; in developed countries, 8% (1). Primary liver cancer (hepatocellular carcinoma [HCC], cholangiocarcinoma, hepatoblastoma, and angiosarcoma [2]), which is among the leading cancers associated with infection (1), ranks sixth among cancers worldwide and is the 18th most common malignancy in the United States (3,4). Notable causes of HCC include chronic infection with hepatitis B or C virus and alcoholic cirrhosis (5).

HCC accounts for 70% to 85% of primary liver cancer in most countries. Rates are highest in eastern Asia, Southeast Asia, and sub-Saharan Africa (6), with the pronounced exceptions of parts of the Philippines and Thailand, where cholangiocarcinoma secondary to liver fluke infestation is common (5). In the United States, primary liver cancer is the fourth most common cancer for males of Asian/Pacific Islander origin and the eighth for males of Hispanic ethnicity (4); the 5-year survival rate is only 8% (7). The incidence rate of HCC in the United States is reportedly higher for blacks, Asians/Pacific Islanders, and Hispanics than for whites (8-11).

Overall, incidence of HCC doubled in the United States from the 1970s through the 1990s (8-11). These findings, which are based mainly on data from 9 of the population-based cancer registries that participate in the National Cancer Institute's (NCI's) Surveillance, Epidemiology, and End Results (SEER) Program, cover approximately 10% of the U.S. population. Unfortunately, information on incidence for population groups other than whites and blacks is limited.

Our study assesses recent HCC incidence rates and trends in the overall U.S. population and among whites, blacks, Asians/Pacific Islanders, and Hispanics. We used data from the Centers for Disease Control and Prevention's (CDC's) National Program of Cancer Registries (NPCR) and the SEER Program.

## Methods

### Data sources

Registries participating in the NPCR and the SEER Program are population-based; combined, they cover 100% of the U.S. population (4). Both programs use the standard data items and codes documented by the North American Association of Central Cancer Registries (NAACCR). As detailed elsewhere (4), hospitals and other facilities collect information from medical records on cancer cases, including patient demographics; a small percentage of cases are identified from death certificates only.

We analyzed data on cancer incidence by year of diagnosis for 1998 through 2003 from registries that met the following data quality criteria for all cancer sites combined for each study year: 1) case ascertainment was at least 90% complete as measured by methods developed by NAACCR, 2) 97% or more cases passed a standard set of computerized edits, 3) 5% or fewer cases were reported by death certificate only or were missing information on the patient's race, and 4) 3% or fewer cases were missing information on the patient's sex or age (4). NPCR and SEER registries augment identification of Hispanic ethnicity with a hierarchical algorithm based on race, birthplace, sex, maiden name, and surname (12). Population denominators for calculating incidence rates were derived from the standard 2000 U.S. Census population (4).

NPCR and SEER registries from 38 states and the District of Columbia met our inclusion criteria. These registries covered approximately 83% of the overall U.S. population and 84% of whites, 74% of blacks, 90% of Asians/Pacific Islanders, and 91% of Hispanics. NPCR data used for this study were reported to CDC as of January 31, 2006. SEER data were reported to NCI as of November 1, 2005. CDC's institutional review board approved this study.

### Variables

To identify HCC cases, we used the *International Classification of Diseases for Oncology, Third Edition* (ICD-O-3) site code for liver (C22.0) and histology codes for HCC (8170, 8172–8175) (13). We excluded fibrolamellar carcinoma (histology code 8171; n = 361), a rare type of liver cancer whose etiology, population distribution, and prognosis differ from those of HCC (14). We also did not include liver cancers with a histology of combined hepatocellular carcinoma and cholangiocarcinoma (histology code 8180; n = 584).

We classified the 38 states in our study and the District of Columbia according to the U.S. Census Bureau's definition of regions (15): 1) Northeast — Connecticut, Maine, Massachusetts, New Jersey, New York, Pennsylvania, Rhode Island, and Vermont; 2) Midwest — Illinois, Indiana, Iowa, Kansas, Michigan, Minnesota, Missouri, Nebraska, Ohio, and Wisconsin; 3) West — Alaska, California, Colorado, Hawaii, Idaho, Montana, New Mexico, Oregon, Utah, and Washington; and 4) South — Alabama, Arkansas, Delaware, District of Columbia, Florida, Kentucky, Louisiana, Oklahoma, South Carolina, Texas, and West Virginia. Study data covered 98% of the population in the Northeast and Midwest, 88% in the West, and 63% in the South.

We tabulated Hispanic ethnicity independent of race, unless otherwise stated. Because work is in progress to improve the accuracy of cancer rates among American Indians/Alaska Natives, we do not present data on this population separately. These data are, however, included in the overall analysis. We classified places of birth outside the United States, Puerto Rico, or the U.S. island areas (American Samoa, the Commonwealth of the Northern Mariana Islands, Guam, and the U.S. Virgin Islands) as non-U.S. birthplaces (16). Birthplace for patients diagnosed with HCC during the study period was available from 13 SEER registries (covering 14% of the U.S. population) and from the California statewide NPCR registry (covering 12% of the U.S. population). HCC incidence data for Asians/Pacific Islanders in California were available for the following subgroups: Chinese, Vietnamese, Korean, Filipino, and Japanese (17).

### Analysis

We present annual and average annual incidence rates per 100,000 population. The average annual incidence rate is the total number of cases reported from 1998 through 2003 divided by the sum of the annual denominators. All rates except those that are age-specific were age-adjusted by the direct method to the standard 2000 U.S. Census population on the basis of 19 groups (less than 1 year, 1–4 years, 5–9 years, 10–14, . . . 85 years and older) (4). Age adjustment was performed for results within the age strata less than 45, 45–59, 60–74, and 75 years and older.

We computed 95% confidence intervals (CIs) for the age-adjusted rates by the gamma method (18). We calculated rate ratios (RRs) comparing age-adjusted HCC incidence rates (19) and used the average annual percentages of change (APC) to evaluate trends.

To estimate APC, we fit a linear regression line to the natural logarithm of the age-adjusted annual rates (*r*) with year of diagnosis as the predictive variable: ln(*r*) = *m*(year) + *b*, where *m* is the slope of the regression line and *b* is the y-intercept (20).

We calculated APC from the formula APC = 100(*e^m^* − 1). A positive APC indicates an increasing trend, and a negative APC, a decreasing trend. We assessed statistical significance at α < .05, determined by a 2-tailed test. For the analysis, we used SEER*Stat, version 6.2.4 (National Cancer Institute, Bethesda, Maryland).

## Results

From 1998 through 2003, 48,048 HCC cases were reported to the registries used in our study. In all, 95% of the cases were reported from hospital sources, 3% were identified solely from autopsy records or death certificates, and the remainder from nonhospital sources. The methods of diagnosis included microscopic confirmation (79%), radiography without microscopic confirmation (13%), clinical diagnosis (4%), and unknown (4%).

The average annual age-adjusted incidence rate of HCC was 3.4 per 100,000 population (95% CI, 3.4–3.4) (data not shown). Although whites accounted for three-fourths of HCC cases, they had the lowest rates (Table 1). The age-adjusted incidence rate of HCC for blacks was approximately 1.7 times that for whites, and the rate for Asians/Pacific Islanders was approximately 4 times that for whites. The risk of HCC for Hispanics was approximately 2.5 times that for non-Hispanics. By region, the incidence rate was lowest in the Midwest and highest in the West.

**Figure 1:**
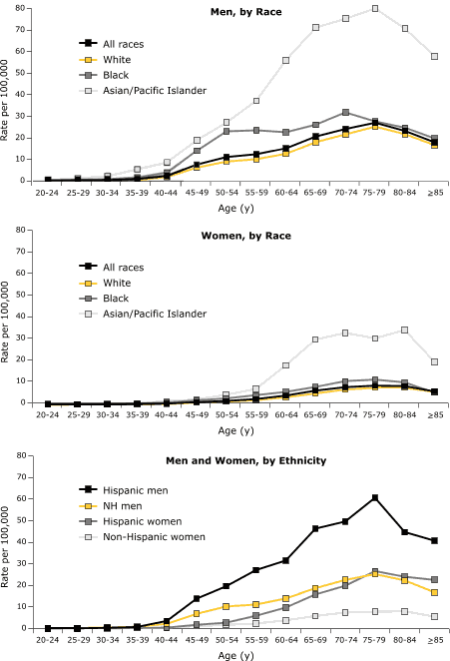
Age-Specific Incidence of Hepatocellular Carcinoma, by Patients' Sex and Race/Ethnicity, United States, 1998–2003.

Overall, the median age at diagnosis was 64 years for males and 71 years for females; this value was lowest for black males and females. By race/ethnicity, the median ages among males were 66 years for whites, 57 for blacks, and 61 for Asians/Pacific Islanders and Hispanics. Among females, median ages were 72 years for whites, 66 for blacks, 68 for Asians/Pacific Islanders, and 70 for Hispanics. Across the age-at-diagnosis groups, average annual incidence was highest for both males and females of Asian/Pacific Islander origin (Figure 1). After age 40 for Hispanic men and age 45 for Hispanic women, age-specific rates were consistently higher than those for non-Hispanics. Black males had an initial steep increase in incidence by age that approximated the rate for Asians/Pacific Islanders; however, the rate for blacks then leveled off and finally, by approximately age 75 years, approached the rate for whites (Figure 1). Overall, the incidence rate for males was 3.7 times that for females (Table 1). The incidence rate for males younger than 45 years was 5 times that for females in that age group, and the rate for men 45–59 years was 5.6 times that of women in that age group.

**Table:** Men, by Race

**Table:** Women, by Race

**Age, y**	**Rate per 100,000**			

	**All races**	**White**	**Black**	**Asian/Pacific Islander**
20-24	0.1	0.1	0.1	0.4
25-29	0.1	0.1	0.1	0.1
30-34	0.1	0.1	0.2	0.5
35-39	0.2	0.2	0.5	0.6
40-44	0.5	0.3	1.0	1.6
45-49	1.2	1.0	2.0	2.4
50-54	1.7	1.4	2.9	4.6
55-59	2.6	2.1	4.4	7.3
60-64	4.2	3.4	5.9	18.2
65-69	6.5	5.2	8.2	30.1
70-74	8.1	7.0	10.9	33.2
75-79	8.8	7.8	11.6	30.7
80-84	8.6	7.8	10.3	34.5
≥85	6.1	5.9	5.5	19.8

**Table:** Men and Women, by Ethnicity

**Age, y**	**Rate per 100,000**			

	**Hispanic men**	**Non-Hispanic men**	**Hispanic women**	**Non-Hispanic women**
20-24	0.1	0.1	0	0.1
25-29	0.1	0.2	0.1	0.1
30-34	0.3	0.4	0.1	0.2
35-39	0.7	0.8	0.2	0.2
40-44	3.5	2.2	0.4	0.5
45-49	13.8	6.8	1.7	1.1
50-54	19.6	10.2	2.7	1.7
55-59	27.1	11.1	5.9	2.3
60-64	31.5	13.9	9.7	3.8
65-69	46.3	18.7	15.8	5.8
70-74	49.6	22.5	19.8	7.4
75-79	60.6	25.2	26.6	7.8
80-84	44.7	22.3	24.0	8.0
≥85	40.7	16.8	22.5	5.5

Data cover 83% of the U.S. population and are from population-based cancer registries that participate in the National Program of Cancer Registries (NPCR) and the Surveillance, Epidemiology, and End Results (SEER) Program and that met study criteria.

### Temporal trends

**Figure 2:**
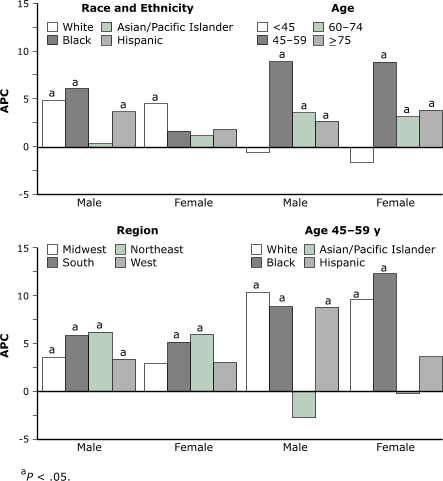
Annual Percentage Change (APC) in Incidence of Hepatocellular Carcinoma, United States, 1998–2003.

The age-adjusted incidence rate for males rose from 5.0 in 1998 to 6.2 in 2003, an increase of 24% (age-adjusted RR, 1.25; *P* < .05) (Table 1). For females, incidence in 2003 was 31% higher than in 1998 (age-adjusted RR, 1.27; *P* < .05). The APC was 4.8% (*P* < .05) for males and 4.3% (*P* < .05) for females. We found significant increasing trends for males in all racial/ethnic subgroups except Asians/Pacific Islanders; for females, only whites had a significant increasing trend (Figure 2). In the specific age groups examined, the largest increases occurred among patients aged 45–59 years at diagnosis (APC 9.0% for men and for women). Among men aged 45–59 years at diagnosis, whites, blacks, and Hispanics had significant increases each year, ranging from 9% to 10%; rates for Asian/Pacific Islander males were statistically unchanged. The pattern by race was similar for women aged 45–59 years at diagnosis, with the exception of the pattern for Hispanics (Figure 2).

**Table:** 

Data cover 83% of the U.S. population and are from population-based cancer registries that participate in the National Program of Cancer Registries (NPCR) and the Surveillance, Epidemiology, and End Results (SEER) Program and that met study criteria.

a P < .05 indicates that the APC is significantly greater than zero.

The median age at diagnosis decreased from 1998 through 2003 (males, from 65 to 62 years; females, from 71 to 70 years), a change that reflects the disproportionate increases in rates among middle-aged people. Also, because rates increased for whites but not for Asians/Pacific Islanders, the RR between these two groups declined over time. For males, the RR between Asians/Pacific Islanders and whites was 4.1 (95% CI, 3.8–4.3) for 1998 through 1999 but dropped for the interval 2002 through 2003 to 3.4 (95% CI, 3.3–3.6). For females, comparable RRs were 4.6 (95% CI 4.2–5.2) and 4.1 (95% CI, 3.7–4.5). In contrast, the RRs between blacks and whites changed very little (data not shown in table).

### Place of birth

Information on place of birth was available for 7457 (78%) of the 9615 patients in the SEER registries. Of these patients, a substantially higher proportion of Asians/Pacific Islanders (84%) than whites (20%) or blacks (7%) was foreign-born (Table 2). Among whites, the proportion with non-U.S. birthplaces was higher for Hispanics (42%) than for non-Hispanics (12%).

### Rates for Asian/Pacific Islander subgroups

Almost one-half of HCC cases among Asians/Pacific Islanders in our study were identified from the California registry. Among males in this group, age-adjusted incidence rates were highest for the Vietnamese subgroup (53.6) and lowest for the Japanese subgroup (8.4) (Table 3). Among females, rates were highest for the Vietnamese (14.3) and Korean (12.3) subgroups and lowest for the Filipino subgroup (3.7). The proportion of foreign-born patients was 95% or higher for all subgroups except Japanese (77%).

## Discussion

Previous studies have indicated that the incidence of HCC in the United States is higher among males than females and higher among blacks and Asians/Pacific Islanders than whites (8-11). We extend these findings by reporting the magnitude of these differences at the national level, and we show that the disparity between Asians/Pacific Islanders and whites decreased over the 6-year study period because rates increased among whites but not among Asians/Pacific Islanders. We also demonstrate that HCC incidence rates vary substantially among U.S. Asian/Pacific Islander subpopulations, with Vietnamese and Korean subgroups having the highest rates. Previous studies comparing rates between Asian/Pacific Islander groups in the United States were based on older data or did not differentiate HCC from other liver cancers (11,17,21-24).

Hepatitis B, a primary cause of HCC, is endemic in Southeast Asia, China, and other Asian countries, where most infections are acquired in early childhood through either mother-to-infant transmission or exposure to chronically infected household members (25). We found that most Asian/Pacific Islander patients with HCC were foreign-born and, therefore, likely to have acquired hepatitis B at birth or during early childhood and to have been infected for many years. Because measures to prevent perinatal hepatitis B transmission did not become available until the 1980s, most U.S.-born Asians/Pacific Islanders in our study most likely acquired their infections in a similar manner (26). We were unable to compute HCC incidence rates by birthplace because population denominators were not available by this variable. However, previous research reports that rates of liver cancer are higher for males born in Asia than for Asian males born in the United States, who in turn have rates higher than those for white males in the United States (21).

The racial/ethnic differences in age-specific incidence and trends for HCC that we found most likely reflect differences in patterns of chronic hepatitis B and hepatitis C as well as host factors and the modulating effects of cofactors such as concomitant infections, alcohol intake, and diabetes mellitus (5,27,28). The limited etiologic data available suggest that hepatitis B has been the major cause of HCC among Asians/Pacific Islanders and that hepatitis C is the leading cause overall and predominates among white, black, and Hispanic patients with HCC in the United States (28-30). These data are for select groups of patients, however, many of whom received a diagnosis of HCC 10 or more years ago. Our ability to ascribe current HCC incidence to different etiologies is very limited. Surveillance for new hepatitis B and C virus infections, which have declined considerably in incidence over the past 2 decades, is of limited relevance because HCC typically develops after a lag time of 2 to 4 decades after hepatitis B or C virus infection (31).

In the United States, national seroprevalence surveys have shown that chronic hepatitis C is approximately 3 times more common than chronic hepatitis B, but the surveys provide limited data by race/ethnicity and region (32,33). Hepatitis C has a marked cohort effect, with prevalence being highest among people born from 1945 through 1964. Most of these people became infected during the 1960s through the 1980s, when incidence was highest, and are now at increased risk of developing chronic liver disease (32,34,35). Much of the recent increase in HCC incidence, and the higher increase in the age group 45–59 years, most likely reflects the aging of these cohorts. Etiologic studies at the individual level are needed to confirm the associations with HCC and to extend previous findings. For this research, various approaches should be explored, including automated capture of available data from electronic health records and other data sources and linkages between the NPCR and SEER programs and registries for chronic hepatitis B and C in states where these registries are well-established.

Our study has certain limitations. First, we were unable to analyze etiology because this information is not captured by the NPCR and SEER registries. Second, information on birthplace from the SEER and California registries may not be generalizable because people with unknown birthplaces are more likely to be born in the United States and because the SEER population tends to be more urban and foreign-born than does the general U.S. population (8,36). Third, because the NPCR program was established recently, we were unable to estimate long-term trends in HCC rates at the national level. Fourth, the population coverage for the South was approximately 63%, and the rates for states that were excluded in this region or elsewhere may differ from those we report here. Fifth, increase in the use of noninvasive methods of diagnosis (e.g., radiography) could result in inclusion of more false-positive cases over time. We obtained similar results, however, when we restricted the analysis to histologically confirmed cases (data not shown). Finally, better reporting of HCC over time might account for the temporal increase. The proportion of all primary liver cancers with poorly specified morphology (histology codes 8000–8140) decreased by 5 percentage points from 1998 (21%) to 2003 (16%). Even if such cases are now being coded as HCC, however, this change can account for only a small portion of the temporal increase, and the variation in trends by age group and race suggests that the increases are not an artifact of better reporting.

Approaches to preventing HCC include primary prevention of viral hepatitis infections and alcohol-related liver disease, secondary prevention of progression to cirrhosis, and prevention of cancer in patients with cirrhosis (37). A strategy of universal infant vaccination with hepatitis B vaccine, the first vaccine developed to prevent cancer, had been adopted in 150 (78%) of the 192 World Health Organization member states by 2003 (25). In Taiwan, reductions in HCC incidence were demonstrated after implementation of infant vaccination programs against hepatitis B virus (38). In the United States, the impact of recommendations from the Advisory Committee on Immunization Practices is reflected in declining hepatitis B incidence, which has been striking since the implementation of universal infant vaccination in 1991 (26,39). Many U.S.-born residents and immigrants, however, already have chronic hepatitis B or C virus infections, so these viruses will most likely persist for decades as major causes of HCC. Consequently, support is needed for secondary prevention efforts, which rely on identification of people with chronic viral hepatitis infection or excessive alcohol consumption to allow for appropriate medical management, including counseling and evaluation for antiviral treatment (40). Furthermore, periodic screening with alpha-fetoprotein and ultrasound has been recommended to promote early detection of HCC among selected populations with evidence of liver disease, but whether this policy results in a reduction in mortality rates has not been evaluated (41).

We documented rising HCC incidence rates in the United States during an era in which the overall incidence of cancer has stabilized (42). The considerable diversity in trends observed among demographic subpopulations most likely reflects differences in primary etiology. As others have observed, over the next decade HCC cases can be expected to continue to increase among people with chronic hepatitis C, but the impact on overall HCC incidence rates is difficult to predict because of limited information on etiology (32,35). Similarly, the impact on HCC incidence rates of immigration of people from countries where hepatitis B is endemic will be hard to assess unless more detailed demographic and etiologic data are available. To better characterize HCC trends and guide the planning and evaluation of prevention programs, representative etiologic data are needed to complement the existing system of cancer surveillance in the United States.

## Figures and Tables

**Table 1:** Demographic Characteristics of People with Hepatocellular Carcinoma (N = 48,048), United States, 1998–2003^a^

Demographic Characteristics	No. Cases (%)		Age-Adjusted Incidence Rate^b^ (95% CI)		Rate Ratio (95% CI)	
	Males	Females	Males	Females	Males	Females
**Overall**	35,859 (100)	12,189 (100)	5.6 (5.6-5.7)	1.5 (1.5-1.6)	NA	NA
**Age at diagnosis, y**						
<45	2,159 (6)	611 (5)	0.5 (0.5-0.5)	0.1 (0.1-0.1)	Ref	Ref
45-59	12,759 (36)	2,339 (19)	10.0 (9.8-10.2)	1.8 (1.7-1.8)	20.92^c^ (19.98-21.90)	13.01^c^ (11.90-14.24)
60-74	13,291 (37)	4,939 (41)	19.7 (19.4-20.1)	6.2 (6.0-6.4)	41.16^c^ (39.33-43.10)	45.97^c^ (42.25-50.08)
≥75	7,650 (21)	4,300 (35)	23.6 (23.1-24.1)	8.1 (7.8-8.3)	49.29^c^ (46.98-51.73)	59.83^c^ (54.95-65.23)
**Race^d^**						
White	26,663 (76)	9,058 (76)	4.8 (4.7-4.9)	1.3 (1.3-1.3)	Ref	Ref
Black	4,521 (13)	1,466 (12)	8.4 (8.1-8.7)	2.1 (2.0-2.2)	1.75^c^ (1.69-1.81)	1.64^c^ (1.55-1.74)
Asian/Pacific Islander	4,011 (11)	1,454 (12)	17.6 (17.0-18.2)	5.6 (5.3-5.9)	3.67^c^ (3.54-3.80)	4.32^c^ (4.08-4.58)
**Ethnicity**						
Non-Hispanic	30,570 (85)	10,308 (85)	5.2 (5.2-5.3)	1.4 (1.4-1.4)	Ref	Ref
Hispanic	5,289 (15)	1,881 (15)	11.4 (11.0-11.7)	3.7 (3.5-3.9)	2.18^c^ (2.12-2.25)	2.62^c^ (2.50-2.76)
**Region**						
Midwest	7,331 (20)	2,585 (21)	4.3 (4.2-4.4)	1.2 (1.2-1.2)	Ref	Ref
South	9,490 (26)	3,192 (26)	5.4 (5.3-5.5)	1.5 (1.4-1.5)	1.26^c^ (1.23-1.30)	1.22^c^ (1.16-1.29)
Northeast	9,278 (26)	2,892 (24)	6.3 (6.1-6.4)	1.5 (1.4-1.6)	1.46^c^ (1.41-1.50)	1.25^c^ (1.19-1.32)
West	9,760 (27)	3,520 (29)	6.9 (6.8-7.0)	2.1 (2.1-2.2)	1.61^c^ (1.56-1.66)	1.79^c^ (1.70-1.89)
**Diagnosis year**						
1998	5,032	1,712	5.0 (4.9-5.1)	1.3 (1.3-1.4)	Ref	Ref
1999	5,251	1,938	5.1 (5.0-5.3)	1.5 (1.4-1.6)	1.02 (0.98-1.07)	1.12^c^ (1.05-1.20)
2000	5,751	1,998	5.5 (5.4-5.6)	1.5 (1.5-1.6)	1.10^c^ (1.06-1.14)	1.14^c^ (1.07-1.22)
2001	6,248	2,037	5.9 (5.7-6.0)	1.5 (1.5-1.6)	1.17^c^ (1.13-1.22)	1.15^c^ (1.08-1.23)
2002	6,594	2,194	6.0 (5.9-6.2)	1.6 (1.6-1.7)	1.21^c^ (1.16-1.25)	1.23^c^ (1.15-1.31)
2003	6,983	2,310	6.2 (6.1-6.4)	1.7 (1.6-1.8)	1.25^c^ (1.20-1.30)	1.27^c^ (1.20-1.36)

NA indicates not applicable; Ref, referent.

a Data cover 83% of the U.S. population and are from population-based cancer registries that participate in the National Program of Cancer Registries and the Surveillance, Epidemiology, and End Results Program and that met study criteria for data for all years.

b Rates are per 100,000 population per year, age-adjusted to the standard 2000 U.S. Census population.

c *P* < .05.

d Data not presented for other races.

**Table 2:** Incidence of Hepatocellular Carcinoma and Birthplaces of Patients, by Race and Hispanic Ethnicity, SEER, 1998–2003

Race/Ethnicity	No. Cases	No. Patients With Known Birthplace (%)	Patients With Non-U.S. Birthplace,^a^ %		
			Males	Females	All
Overall^b^	9615	7457 (78)	34	42	36
White	5779	4445 (77)	18	26	20
Hispanic	1563	1179 (75)	36	56	42
Non-Hispanic	4216	3266 (77)	11	14	12
Black^c^	1110	865 (78)	6	10	7
Asian/Pacific Islander^c^	2578	2056 (80)	84	85	84

SEER indicates Surveillance, Epidemiology, and End Results Program.

a Among patients with known birthplace.

b Data for American Indians/Alaska Natives, although not presented individually, are included in the “overall” category.

c Fewer than 1% of black and Asian/Pacific Islander patients are of Hispanic ethnicity.

**Table 3:** Incidence of Hepatocellular Carcinoma and Birthplaces of Patients Among Selected Asian/Pacific Islander Subgroups, California, 1998–2003

Asian/Pacific Islander Subgroup	No. Cases	Age-Adjusted Rate (95% CI)^a^			Patients With Non-U.S. Birthplace,^b^ %		
		Males	Females	All	Males	Females	All
Vietnamese	602	53.6 (48.2-59.7)	14.3 (11.8-17.3)	32.7 (29.9-35.7)	100	100	100
Korean	372	31.5 (27.4-36.3)	12.3 (10.2-14.9)	20.5 (18.4-22.9)	97	97	97
Chinese	781	20.8 (19.1-22.6)	6.5 (5.6-7.4)	13.1 (12.2-14.0)	95	95	95
Filipino	410	14.2 (12.6-15.9)	3.7 (3.0-4.5)	8.1 (7.3-8.9)	98	96	98
Japanese	206	8.4 (6.7-10.5)	6.8 (5.6-8.3)	7.7 (6.7-8.9)	61	87	77

a Rates are per 100,000 population per year and age-adjusted to the standard 2000 U.S. Census population.

b Among patients with known birthplaces (79% of males, 81% of females).
